# Identification of Bilateral Changes in TID1 Expression in the 6-OHDA Rat Model of Parkinson's Disease

**DOI:** 10.1371/journal.pone.0026045

**Published:** 2011-10-07

**Authors:** Juliane Proft, Jamshid Faraji, Jerrah C. Robbins, Fabiola C. R. Zucchi, Xiaoxi Zhao, Gerlinde A. Metz, Janice E. A. Braun

**Affiliations:** 1 Hotchkiss Brain Institute, Department of Physiology and Pharmacology, University of Calgary, Calgary, Canada; 2 Canadian Centre for Behavioural Neuroscience, University of Lethbridge, Lethbridge, Canada; 3 Neuroscience Research Centre, Golestan University of Medical Sciences, Gorgan, Islamic Republic of Iran; National Institutes of Health, United States of America

## Abstract

Parkinson's disease (PD) is a common neurodegenerative disease characterized by the loss of dopaminergic neurons in the substantia nigra and the aggregation of α-synuclein into Lewy bodies. Existing therapies address motor dysfunction but do not halt progression of the disease. A still unresolved question is the biochemical pathway that modulates the outcome of protein misfolding and aggregation processes in PD. The molecular chaperone network plays an important defensive role against cellular protein misfolding and has been identified as protective in experimental models of protein misfolding diseases like PD. Molecular mechanisms underlying chaperone-neuroprotection are actively under investigation. Current evidence implicates a number of molecular chaperones in PD including Hsp25, Hsp70 and Hsp90, however their precise involvement in the neurodegenerative cascade is unresolved. The J protein family (DnaJ or Hsp40 protein family) has long been known to be important in protein conformational processes.

We assessed sensory and motor function of control and PD rats and then evaluated the brain region-specific expression levels of select J proteins by Western analysis. Surprisingly, we observed a widespread 26 kDa breakdown product of the J protein, TID1, (tumorous imaginal discs, mtHsp40 or DnaJ3) in a 6-hydroxydopamine (6-OHDA) rat model of PD in which food handling, gait symmetry and sensory performance were impaired. Greater behavioral deficits were associated with lower TID1 expression. Furthermore, direct application of either 6-OHDA or MPP^+^ (1-methyl-4-phenylpyridinum) to CAD (CNS-derived catecholinaminergic neuronal cell line) cell cultures, reduced TID1 expression levels.

Our results suggest that changes in cellular TID1 are a factor in the pathogenesis of PD by impeding functional and structural compensation and exaggerating neurodegenerative processes. In contrast, no changes were observed in CSPα, Hsp40, Hsp70, Hsc70 and PrP^C^ levels and no activation of caspase3 was observed. This study links TID1 to PD and provides a new target for therapeutics that halts the PD progression.

## Introduction

Parkinson's disease (PD), a neurodegenerative disease that afflicts 1% of the population over 65, is characterized by degeneration of dopaminergic neurons in the substantia nigra and formation of intracytoplasmic -synuclein aggregates called Lewy bodies [1]. Mutations in α-synuclein, DJ-1, PINK-1 and Parkin as well as toxins like 6-hydroxydopamine (6-OHDA), rotenone, paraquat and 1-methyl-4-phenyl 1,2,3,6 tetrahyrdopyridine (MPTP) lead to parkinsonism/PD suggesting that degeneration involves a complex and multifaceted pathway [2], [3]. Clinical symptoms of PD include rigidity, bradykinesia, resting tremor and postural instability. As the disease progresses, patients may also develop serious cognitive decline, which is thought to be causally linked to Lewy body pathology [4]. There is no known cure to prevent or reverse the progression of PD. In this study we begin to address molecular changes underlying PD by evaluating expression levels of putative neuroprotective proteins in PD rats rigorously evaluated for sensorimotor behavior.

Although evidence has suggested a key role of α-synuclein aggregation in the pathology of PD, its exact role is still under debate [5]. Downstream of α-synuclein misfolding the pathogenic sequence of events have been difficult to construe, but studies have shown that abnormal α-synuclein aggregation is linked to altered lipid metabolism that leads to mitochondrial dysfunction in dopaminergic neurons [6], [7]. Dysfunction of an early-acting chaperone in the pathogenic sequence of events could possibly have a toxicity-initiating role while reduced levels and activities of late-acting molecular chaperones in the pathogenic sequence may undermine protection and recovery from cellular damage associated with protein misfolding. While enhancement of molecular chaperone expression has been reported to protect against α-synuclein toxicity [8], many questions remain regarding the role of chaperones in PD progression. One family of co-chaperones known as J proteins are an evolutionarily conserved family (aka DnaJ/Hsp40 family), each member containing a 70 amino acid tetrahelical J domain required for stimulating the ATPase activity of Hsc70 (heat shock cognate protein of 70 kDa) [9]. J proteins adapt Hsc70 for specialized folding tasks and seem to possess decisional power on whether to direct client proteins toward maturation/refolding or degradation. Malfunction of J proteins may therefore be pivotal in the failure of protein homeostasis and neurodegeneration observed in PD.

We tested elements of this hypothesis by evaluating expression levels of select J proteins in a rat model of neuronal death by nigrostriatal injection of 6-OHDA. 6-OHDA exposure was chosen as a PD mimetic in our study because the 6-OHDA model has been extensively characterized functionally [10]. The 6-OHDA-lesion can be compared to other PD mimetics, involves changes in gene transcription [11], and is exaggerated by stress [12]. We identified a 26 kDa immunoreactive product of TID1, a 40/43 kDa J protein in the rat model of PD suggesting that TID1-mediated stability and folding is compromised in PD.

TID1, the mammalian homologue of the *Drosophila* tumor suppressor Tid56, is proposed to have a role in multiple signal transduction processes including clustering of acetylcholine receptors at the neuromuscular junction [13] nerve growth factor-induced neurite outgrowth [14], apoptosis [15], and mitochondrial translocation of proteins [16]. TID1 was first identified as a viral interacting protein [17]. While TID1 knockout mice are embryonic lethal [18], mutations in TID1 have been identified in glioma cell lines [15], indicative of an involvement of TID1 in cell survival pathways. Mammals express two alternatively spliced forms of TID1: TID1-long (43 kDa) and TID1-short (40 kDa). Both isoforms have an amino-terminal mitochondrial targeting sequence, a J domain, a glycine/phenylalanine linker region, a central cysteine-rich region resembling a zinc finger repeat and a carboxy terminal region with some similarity to DnaJA1/DnaJA2. J domains are comprised of four α helices with a highly conserved tripeptide of histidine, proline and aspartic acid located between helices II and III and are found in all members of the J protein family. Via their J domains, J proteins interact with and activate the Hsp70/Hsc70 family of chaperones, which is thought to have a substantive role in PD pathology [19]–[23]. TID1 localizes to mitochondria as well as cytosol supporting the hypothesis that TID1 is important for protein import to the mitochondria [16], [24].

The Hsp70/Hsc70 family has a critical function in a range of cellular processes including the folding of newly synthesized proteins and the rescue of misfolded proteins [25], [26]. Hsp70 colocalizes with Lewy bodies [23]. In PD fly models [19] and in neuroglioma cells [20], overexpression of Hsp70 has been shown to reduce α-synuclein toxicity. In particular, Hsp70 inhibits α-synuclein fibril assembly [21], and reduces α-synuclein aggregates *in vivo* [27]. Inhibition of Hsp70 by BAG5, a BAG domain-containing protein, is reported to lead to dopaminergic neuron death in an *in vivo* model of PD disease [28]. In contrast, it has been reported that Hsp27 but not Hsp70 has a protective effect on α-synuclein-induced toxicity in a rat model of Parkinson disease [29]. In addition, when human α-synuclein containing a point-mutation and Hsp70 are co-overexpressed, Hsp70 was not observed to have a beneficial effect on behavior and α-synuclein aggregation *in vivo* [30]. Conformational work by the Hsp70/Hsc70 family is subject to a multitude of regulators [9], [25], and incompatible findings regarding the role of Hsc70/Hsp70 in PD may well be due to such regulatory cofactors, however further investigation is required.

It has remained untested if members of the J protein family have a substantive role in PD. In order to begin to understand the role that J proteins have in PD we have evaluated the levels of select J proteins in a 6-OHDA rat model of PD. Rats received a unilateral 6-OHDA lesion of the nigrostriatal bundle and were tested in a behavioral test battery comprising a new scoring system for skilled forelimb use, gait symmetry and somatosensory function. 27–33 days following 6-OHDA exposure we observed lower molecular weight (26 kDa) immunoreactive degradation products of the J protein TID1 implicating changes in TID1 activity in degeneration associated with PD. This change was specific to the J protein TID1 in that neither the stress-induced J protein, Hsp40 (heat shock protein of 40 kDa), nor the anti-neurodegenerative J protein, CSPα (cysteine string protein), expression levels were altered in PD rats. The 26 kDa TID1 entity was observed outside the site of 6-OHDA application. It has been generally accepted that TID1 is involved in tumor suppressor activities and loss of TID1 promotes carcinogenesis, here we link TID1 to PD. We speculate that the widespread, bilateral alterations of TID1 would impair cellular ability to maintain mitochondrial protein homeostasis and TID1-specific signaling pathways.

## Results and Discussion

### PD 6-OHDA lesion rats show severe sensorimotor deficits

**Figure 1:**
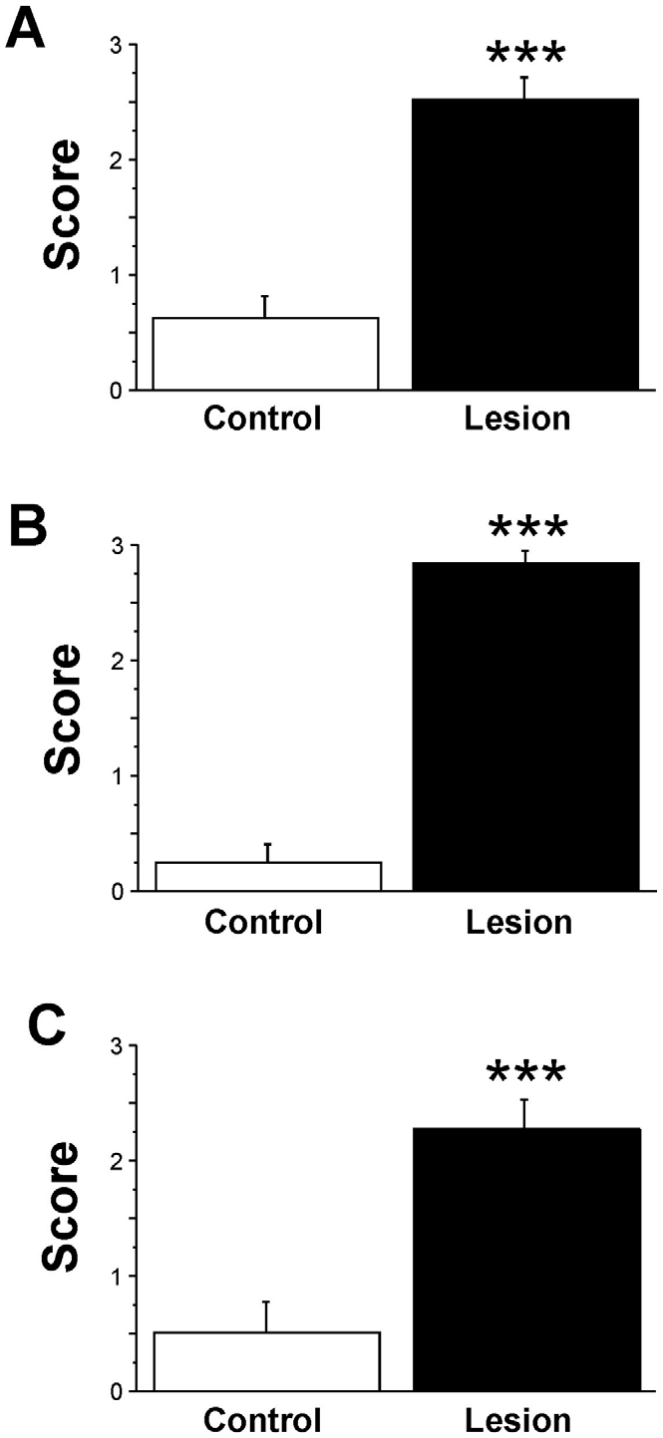
The performance of rats is influenced by 6-OHDA-lesions. (**A**) Sensory Impairment test, (**B**) Gait Symmetry Impairment test (**C**) Food Handling Impairment test in 6-OHDA lesion and saline control rats. Following left hemisphere injection of 2 µl of 6-OHDA or saline, rats were tested for behavioral deficits. A higher behavioral score indicates greater behavioral deficit. *** indicates significance p<0.001.

To begin to address the hypothesis that depletion of neural chaperones contributes to PD pathology, we have examined the levels of the J proteins TID1, CSPα and Hsp40 in the 6-OHDA rat model of PD. Administration of 6-OHDA, a toxic metabolite of dopamine found in the brain and urine of PD patients, is known to produce selective destruction of catecholaminergic neurons. Behavioral tests were performed at 21–26 days following left nigrostriatal 6-OHDA infusion, a time at which maximum dopamine depletion can be expected. Behavioral tests assessed various aspects of sensorimotor function in order to complement the comprehensive molecular assessment. In all tests, rats demonstrated significant deficits in motor and somatosensory function (**Figure 1**). Specifically, while control rats reliably responded to the tactile stimulation (mean score 0.63+/−0.52), 6-OHDA lesion rats were rarely responsive to stimulation (2.56+/−0.63 t = 7.51, p<0.0001; **Figure 1A**). These findings indicate severe somatosensory deficits in 6-OHDA lesion animals.

Furthermore, 6-OHDA lesion rats showed significant gait asymmetry (**Figure 1B**). While controls did not show spontaneous preference when turning (mean score 0.25+/−0.46), 6-OHDA lesion animals (2.94+/−0.25) without exception showed a bias towards the ipsilateral-to-lesion side when turning (t = −18.65, p<0.001). These scores indicate that 6-OHDA lesion rats showed severe motor asymmetry in their stepping pattern leading to ipsilesional turns.

Skilled movement in 6-OHDA lesion rats was also tested and revealed significant differences to control animals (**Figure 1C**). Lesion rats displayed significant impairment in handling and eating pieces of uncooked pasta. Compared to controls (mean score 0.5+/−0.76), 6-OHDA lesion animals (2.31+/−0.95) on average needed four times longer to consume a single piece of spaghetti (t = 4.7, p<0.0001). The time measurement indicates difficulty in distal limb and digit use when handling the pasta in addition to orofacial impairments while chewing.

The behavioral findings in 6-OHDA rats mimic the motor and sensory deficits typically displayed in human PD. After behavioral testing was completed the striatum, midbrain, cortex, prefrontal cortex, cerebellum and hippocampus were dissected from right and left hemispheres 27–33 days following injection, solubilized and subjected to Western analysis.

### PD 6-OHDA rats show widespread appearance of a 26 kDa TID1 immunoreactive species

**Figure 2:**
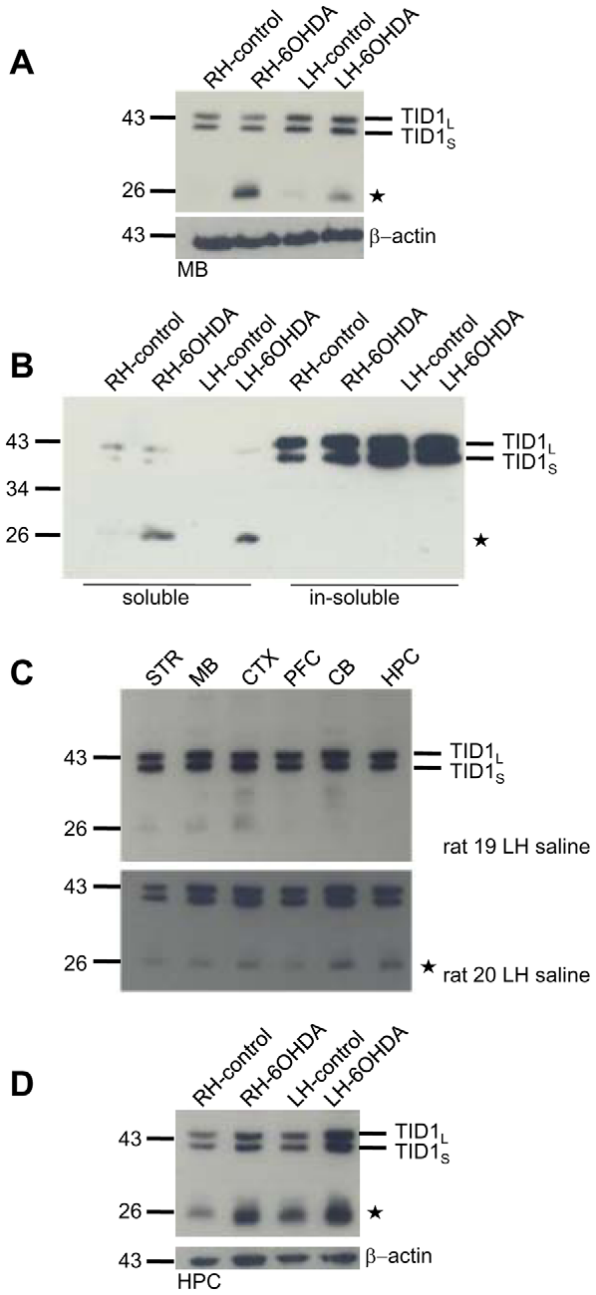
Unilateral 6-OHDA-lesions increased TID1 (DnaJA3) chaperone breakdown. (**A**) Following left hemisphere injection of 2 µl of 4 mg/ml 6-OHDA or saline, rats were tested for neurodegeneration and the indicated brain region were dissected 27–33 days following injection. 30 µg of solubilized midbrain was heated at 95°C for 5 min, resolved by SDS-PAGE, transferred to nitrocellulose and probed with anti-TID1 monoclonal antibody. The Western blot shown is representative of 11 6-OHDA/saline pairs of rats. Actin is shown as a loading control. (**B**) Midbrain samples were fractionated into soluble and insoluble and subjected to Western analysis. (**C**) TID1 expression in the indicated regions of saline injected rats was evaluated by Western analysis with anti-TID1 monoclonal antibody and quantitated by Quantity One (BioRad). **(D)** TID1 expression in 30 µg of solubilized hippocampus. The Western blot shown is representative of 11 6-OHDA/saline pairs of rats. Actin is shown as a loading control.

An abundant bilateral 26 kDa immunoreactive product of the J protein, TID1 was observed in the midbrain of 6-OHDA but not control rats (LH midbrain 3.2 fold increase n = 6) (**Figure 2**). In contrast, the 43 kDa TID1_long_ (TID1_L_) and 40 kDa TID1_short_ (TID1_S_) isoforms were clearly observed in midbrain isolated from the right and left hemisphere of both the saline and 6-OHDA treated animals. Both apoptotic and anti-apopotic activities have been reported for TID1. Specifically, overexpression of TID1_L_ is reported to increase apoptosis induced by mitogen C and TNFα, while TID1_S_ over expression is observed to reduce apoptosis by these agents [31]. It is interesting that a 38 kDa TID1 splice variant with limited expression has been described [32], [33] and found to be highly expressed in two leukemia cell lines [34].

The 26 kDa immunoreactive product is most likely a breakdown product of one of the splice variants of TID1. Since we did not detect the 38 kDa TID1 splice variant in any brain regions evaluated from either control or 6-OHDA rats, we predict that the 26 kDa TID1 derives from the 43/40 kDa TID1 variants. Despite the increase in the 26 kDa immunoreactive band, no reduction in midbrain expression levels of either TID1_L_ or TID1_S_ was observed and no change in the ratio of the two isoforms TID1_L_ and TID1_S_ were detectable indicating that TID1 levels are maintained in the PD model. Actin is shown as a loading control. Even though TID1 has an N terminal mitochondrial targeting sequence, it is known to localize to both the mitochondria as well as transiently in the cytosol [24] relatively less is known about the cellular role of the cytosol to mitochondria transition of TID1. Therefore, we fractionated (30 min 21,000Xg) midbrain lysates to establish if the 26 kDa TID1 co fractionated with TID1_L,_-_S_ (**Figure 2B**). TID1_L_,-_S_ was found in the insoluble (1% TX100, 0.1%SDS) fraction while the 26 kDa product was present in the soluble fraction. Although the monoclonal antibody utilized was generated against full-length human TID1 and the epitope of the antibody is not reported, the antibody does not distinguish between 40/43 kDa TID1 indicating the antibody does not target the C terminal 27 amino acid region unique to the 43 kDa variant of TID1. We speculate that increases in mitochondrial permeability and/or reduction in translocation of TID1 to the mitochondria may well lead to generation of soluble 26 kDa TID1. In fact, 6-OHDA has been shown to increase mitochondrial permeability [35] and the cytosolic residency time and half life of TID1 has been proposed to be influenced by interactions with cytosolic proteins [24] supporting these ideas.

**Figure 3:**
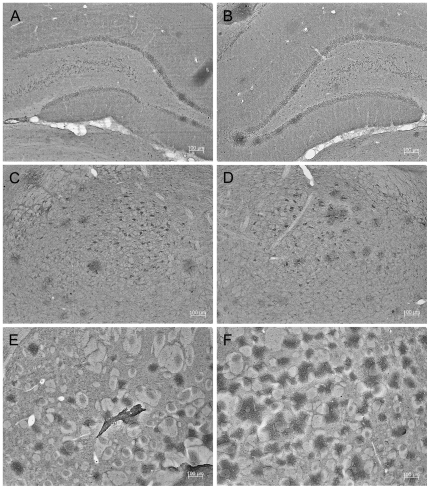
TID1 is widely expressed in brains of 6-OHDA-lesioned rats. TID1 was widely detected by immunohistochemistry in frozen rat brain serial sections. (**A**) Hippocampus: non-lesion hemisphere. (**B**) Hippocampus: lesion hemisphere. (**C**) Substantia nigra: non-lesion hemisphere. (**D**) Substantia nigra: lesion hemisphere. (**E**) Striatum: non-lesion hemisphere. (**F**) Striatum: lesion hemisphere. Scale: 100 µm.

**Figure 2C** demonstrates that in control rats TID1 is widely expressed in striatum, midbrain, cortex, prefrontal cortex, cerebellum and hippocampus. A darker exposure (bottom panel) shows that the 26 kDa immunoreactive TID1 product is weakly detectable in striatum, midbrain, cortex, prefrontal cortex, cerebellum and hippocampus of control rats with the strongest immunoreactivity found in the hippocampus. **Figure 2D** shows the 26 kDa TID1 breakdown product is observed in the hippocampus of both saline as well as 6-OHDA-treated rats, but 3–4 fold higher in 6-OHDA-treated rats. Actin is shown as a loading control. Figure 3 clearly shows that TID1 expression is widespread in hippocampus, substantia nigra and striatum in 6OHDA lesion and non lesion hemisphere of PD rats consistent with western analysis.

**Figure 4:**
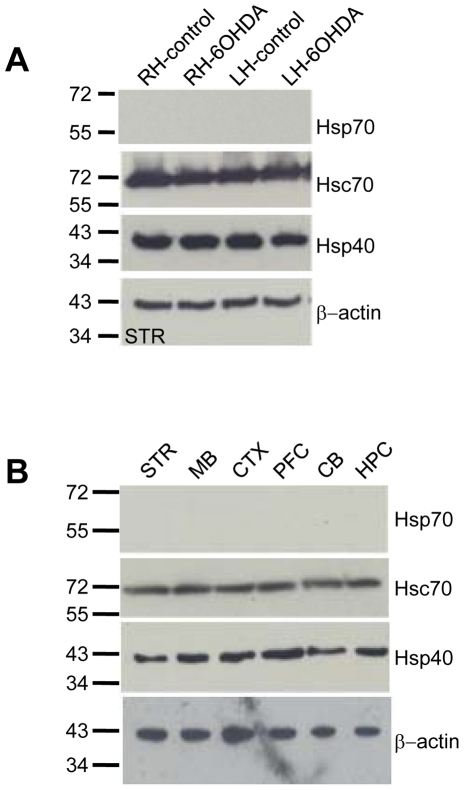
Unilateral 6-OHDA-lesions do not induce the heat shock chaperones Hsp70 and Hsp40. (**A**) Following left hemisphere injection of 2 µl of 4 mg/ml 6-OHDA or saline, rats were tested for neurodegeneration and the expression level of Hsc70, Hsp70 and Hsp40 in the striatum of saline (control) or 6-OHDA lesion rats was determined by Western analysis. **(B**) Western blot showing expression of the indicated chaperones in the striatum (STR), midbrain (MB), cortex (CTX), prefrontal cortex (PFC), cerebellum (CB) and hippocampus (HPC) of saline injected rats were evaluated with indicated antibodies and quantified by Quantity One (BioRad).

In response to a range of stressful stimuli including hyperthermia, a conserved cellular program called the heat shock response is activated and the expression of several chaperones is induced that enhances cell survival. Therefore we next evaluated fractions from control and 6-OHDA rats for the possible induction of stress induced chaperones. **Figure 4A** shows that no differences were observed in expression of either Hsp70 or Hsp40 in the striatum of 6-OHDA compared to control rats. Actin is shown as a loading control. Hsp70 expression was not observed in any brain region of either PD or control rats, (**Figure 4A & B** **, and data not shown**). Furthermore, the constitutively expressed isoform, Hsc70, is abundantly expressed in striatum and its expression does not change in 6-OHDA rats. Hsp40 is endogenously expressed and no further elevation in Hsp40 levels is observed in 6-OHDA animals. Like the striatum, no change in Hsp70, Hsp40 and Hsc70 were identified in any brain regions examined (data not shown).

Taken together these data identify a widespread cytosolic 26 kDa breakdown product of the constitutively expressed J protein, TID1, in the rat neurotoxin model of PD and suggest that TID1 may be involved in altered mitochondrial dynamics in PD progression. In contrast, we found no difference in the expression of the protective heat shock chaperones, Hsp70 and Hsp40, indicating that the critical neuroprotective stress-induced chaperones are not induced in PD 27–33 days after onset. Moreover, no difference in expression levels of the constitutive chaperone Hsc70 between 6-OHDA and control rats was found, indicating the Hsc70 chaperone reservoir is not depleted. Our identification of the breakdown products of the mitochondrial localized J protein TID1 but not the stress-induced J protein Hsp40, is consistent with the recognition of a critical role for mitochondria in inherited forms as well as neurotoxin-induced forms of PD.

### Unilateral 6-OHDA-lesions increases α-synuclein expression but does not alter CSPα expression

**Figure 5:**
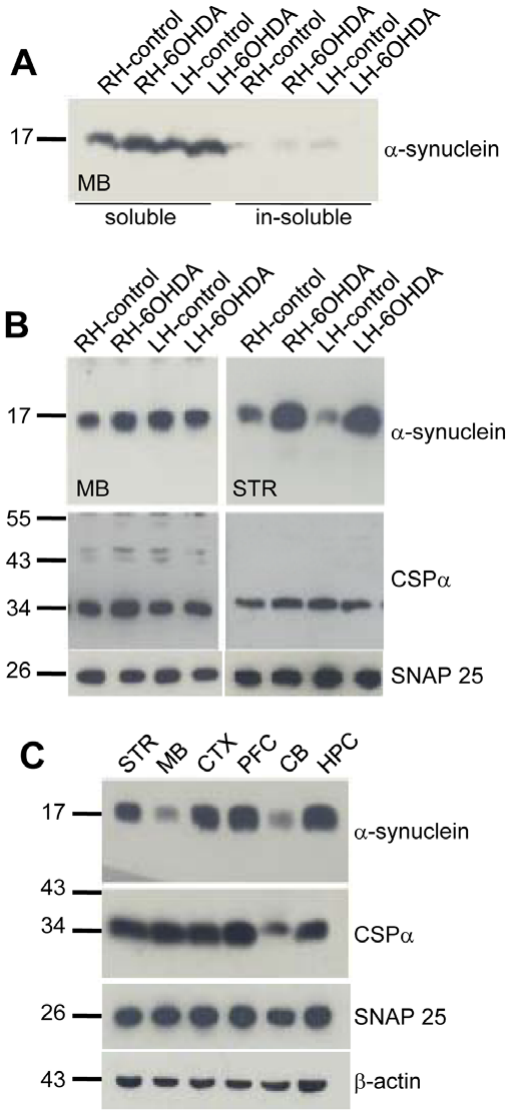
PD rats show increased levels of α-synuclein but not CSPα. Following left hemisphere injection of 2 µl of 4 mg/ml 6-OHDA, rats were tested for neurodegeneration and were dissected 27–33 days following injection. (**A**) 100 µg of total midbrain was fractionated, resolved by SDS-PAGE, transferred to nitrocellulose and α-synuclein was evaluated by Western blot. **(B**) α-synuclein, CSPα and SNAP25 expression levels of saline (control) or 6-OHDA lesion rats in the midbrain and striatum were evaluated by Western analysis using the monoclonal anti-α-synuclein antibody, the monoclonal anti-α-SNAP25 antibody or the anti-CSPα polyclonal antibody. **(C**) Western analysis of α-synuclein, CSPβ and SNAP25 for striatum, midbrain, cortex, prefrontal cortex, cerebellum and hippocampus of the left hemisphere of saline induced rats. Actin is shown as a loading control.

Next we determined levels of α-synuclein in the PD rat model. The exact function of α-synuclein, a cytosolic neuronal protein that localizes with synaptic vesicles as well as the nucleus [36] is not established, however its misfolding is a critical determinant in PD [1]. α-synuclein levels correlate with synaptic function [37]. In midbrain of either 6-OHDA or control treated rats, α-synuclein located to the soluble fraction (**Figure 5A**). **Figure 5B** shows that while α-synuclein expression is higher in the striatum of 6-OHDA rats it is not altered in other brain regions (5B right panel & data not shown). Interestingly, α-synuclein exerts a neuroprotective action by preventing neurodegeneration caused by deletion of the presynaptic J protein cysteine string protein (CSPα) [38]. CSPα is a synaptic vesicle anchored J protein with neuroprotective properties [39], [40]. Therefore, we next examined the possibility that CSPα levels were reduced in brain regions of 6-OHDA compared to saline treated rats. The expression level of CSPα was not altered in 6-OHDA rats. CSPα forms dimers, a process that is modified by heat shock [41] as well as the flavonoid quercetin [42], no differences in CSPα dimerization were found between control and PD rats. **Figure 5C** shows that CSPα levels are high in all brain regions examined with lowest expression in the cerebellum and that α-synuclein levels are also low in cerebellum. While CSPα-knockout mice show approximately 50% decrease in SNAP25 (synaptosomal-associated protein of 25 kDa) levels, a core component of the secretory machinery [38], [43], CSPα and SNAP25 were not altered in the toxin triggered PD rats (**Figure 5C&D**) suggesting that the mechanism(s) of degeneration in CSPα null mice and PD rats are distinct. Actin is shown as a control. Taken together, in PD rats there are widespread, bilateral changes in the J protein TID1 and a localized increase in α-synuclein. No change in the levels of the neuroprotective J proteins CSPα or Hsp40 or the downstream SNAP25 target was observed in PD rats.

### Cellular prion protein levels do not change in a rat model of PD

**Figure 6:**
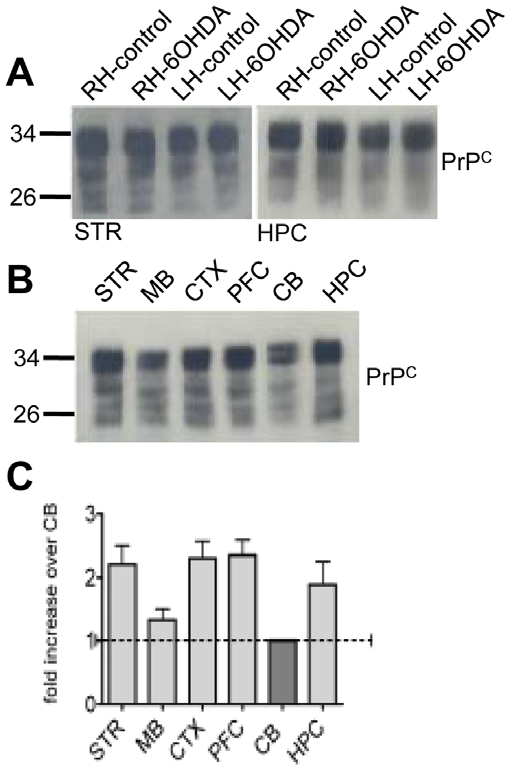
Cellular prion protein (PrP^C^) does not change in PD rats. Following left hemisphere injection of 6-OHDA, rats were dissected 27–33 days post-lesion and 30 µg of homogenate isolated from the indicated brain regions was evaluated by Western analysis with the monoclonal antibody 6H4 (Prionics). (**A**) PrP^C^ expression in right and left striatum and hippocampus of saline and 6-OHDA lesion rats. (**B**) Western analysis of PrP^C^ in the striatum (STR), midbrain (MB), cortex (CTX), prefrontal cortex (PFC), cerebellum (CB) and hippocampus (HPC) of the left hemisphere of saline induced animals. (**C**) Quantification via Quantity One (BioRad) of the Western analysis. The quantification was carried out for eight individual saline treated rats.

Several studies have suggested a protective function for cellular prion protein (PrP^C^) [44]–[47], [47]–[56] and it follows that changes in PrP^C^ expression might influence progression of PD. Nonetheless, deletion of PrP^C^ was not found to alter the disease phenotype in the α-synuclein transgenic mouse model of PD [57]. We therefore assessed PrP^C^ levels in brain regions of the 6-OHDA rat model. **Figure 6A** shows that no detectable changes in PrP^C^ levels were found in striatum and hippocampus in 6-OHDA compared to control rats. No differences were observed in other regions (data not shown). In the control rats, PrP^C^ is widely expressed with lower levels in the midbrain and cerebellum (**Figure 6B and C**).

Taken together, these data demonstrate that, no change is found in expressed levels of the neuroprotective J protein, CSPα, the heat shock proteins or cellular prion protein in the rat neurotoxin model of PD. Although, these results are mainly negative we report them because they address the critical question of the role that these proteins have in PD progression.

### 6-OHDA and MPP^+^ reduce TID1 expression in CAD cells

**Figure 7:**
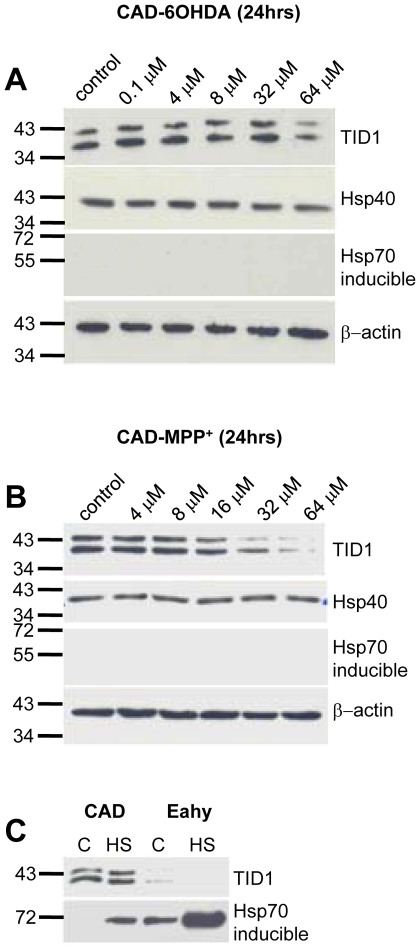
6-OHDA and MPP^+^ reduce TID1 levels in CAD cells . Western analysis of TID1 following (**A**) 6-OHDA or (**B**) MPP^+^ treatment for 24 hrs of CAD cells or Eahy cells. Expression levels of TID1, Hsp40 and Hsp70 were detected using the indicated antibodies. Actin staining is shown as a loading control (**C**) Western analysis of TID1 levels in CAD and Eahy929 control cells following Heat shock treatment (HS).

Since the widespread increase in levels of the 26 kDa TID1 at 27–33 days following 6-OHDA may reflect either neurotoxicity or compensatory changes in the PD rat model, we therefore evaluated TID1 levels in CAD (CNS-derived catecholinaminergic neuronal cell line) cells acutely treated with the neurotoxins 6-OHDA and MPP^+^ (1-methyl-4-phenylpyridinium). **Figure 7A** demonstrates that 64 µM 6-OHDA selectively reduced TID1_L,_-_S_ expression in CAD cells. Furthermore, 16 µM MPP^+^, a mitochondrial complex I inhibitor that compromises mitochondrial integrity and causes Parkinsonism in primates, also reduced TID1_L,_-_S_ expression in CAD cells and did not induce heat shock chaperones (**Figure 7B**). The 26 kDa TID1 product was not observed in either control or toxin-treated neuroblastoma cells. Like that found in intact animals, heat shock chaperones were not induced following neurotoxin treatment. When CAD neuroblastoma cells and Eahy endothelial cells were provided with a conditioning heat shock (40 min at 42°C), Hsp70 was induced while TID1 remained unchanged (**Figure 7C**), confirming TID1 and the TID1 26 kDa product is not a component of the heat shock response. Eahy cells constitutively express Hsp70 which is upregulated in response to heat shock. Our results show that levels of TID1 are acutely susceptible to 6-OHDA and MPP^+^ treatment. In response to either 6-OHDA or MPP^+^ a reduction in TID1_L,_-_S_ levels is observed in cultured cells while *in vivo* localized 6-OHDA treatment initiates a bilateral, widespread 26 kDa TID1 product. While direct application of 6-OHDA to cultured cells triggered acute loss of cellular TID1, in contrast *in vivo* application to axons in the nigrostriatal bundle triggered widespread appearance of the 26 kDa immunoreactive species, suggesting that compensatory mechanisms may be involved. The differences observed between PD rats and neuroblastoma cell lines are under continuing investigation, that said, toxin-induced TID1 changes are clear in both experimental models.

**Figure 8:**
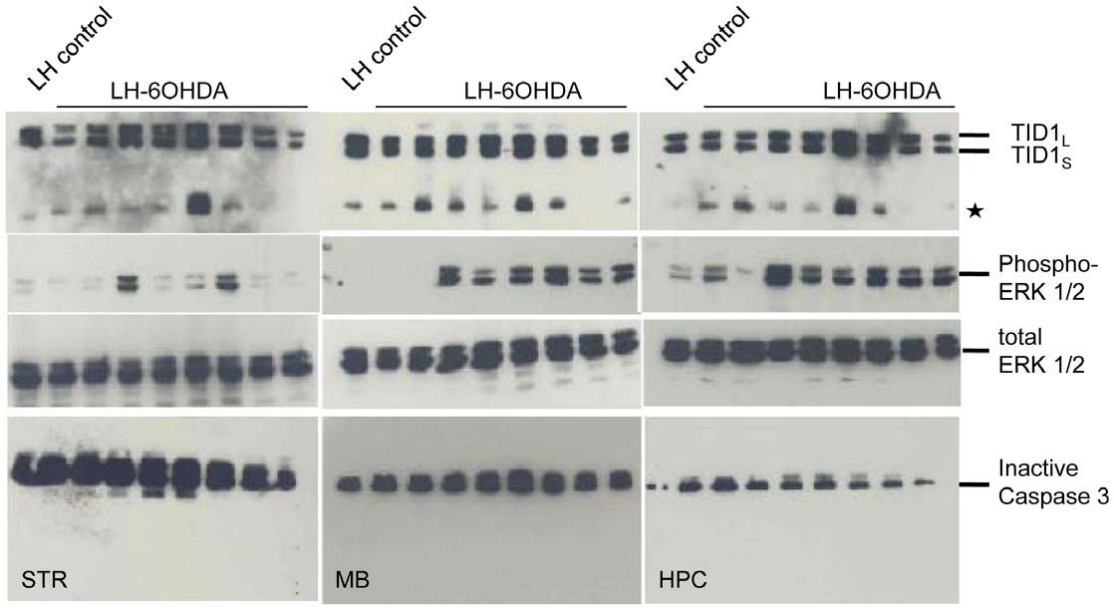
6-OHDA treatment causes ERK phosphorylation and does not activate the Caspase pathway. 27–33 days following left hemisphere injection of 6-OHDA, brain regions were dissected. 30 µg of homogenate isolated form the striatum (STR), midbrain (MB) and hippocampus (HPC) were heated at 95°C for 5 min., resolved by SDS-PAGE, transferred to nitrocellulose membrane and probed for TID1, phosphorylated ERK (phosphorylated p44/42 MAPK), total ERK expression (total p44/42 MAPK) and caspase 3. Lanes from left to right are: control (rat 18), 6-OHDA rats 1, 2, 3, 6, 7, 12, 13, and 16.

During the course of these experiments we discovered that the levels of the 26 kDa TID1 fragment were variable between PD rats. For example, Rat #7 displayed smaller behavioral impairments associated with higher presence of TID1 fragment in the striatum, midbrain and hippocampus. By contrast, rat #16 showed greater behavioral impairments associated with lower presence of TID1 in any of the regions. **Figure 8** (top panel) shows the 26 kDa TID1 fragment in the striatum, midbrain and hippocampus of 8 separate 6-OHDA rats. The 26 kDa TID1 fragment was more abundant in some rats. Since TID1 is implicated in multiple signaling pathways (e.g. cell proliferation, and apoptosis) and over expression of TID1 has been shown to enhance ERK/MAP kinase activation [14], we evaluated the phosphorylated ERK levels in the 6-OHDA rats, to establish if the 26 kDa fragment correlated with increased ERK activation. Many 6-OHDA rats showed increased levels of phosphoERK and this increase in phosphorylation did not correlate with changes in total ERK levels (**Figure 8**). The 6-OHDA rats with the highest levels of the 26 kDa fragment did not show the greatest ERK activation. The lower panel shows that there is no activation of caspase 3 in any animals. These data lead us to conclude that in 6-OHDA rats, both the 26 kDa TID1 product and activation of the ERK/MAP-Kinase pathway correlate with dopamine depletion, however cellular levels of the 26 kDa TID1 product do not correlate with levels of ERK phosphorylation at the 27–33 day time point.

**Figure 9:**
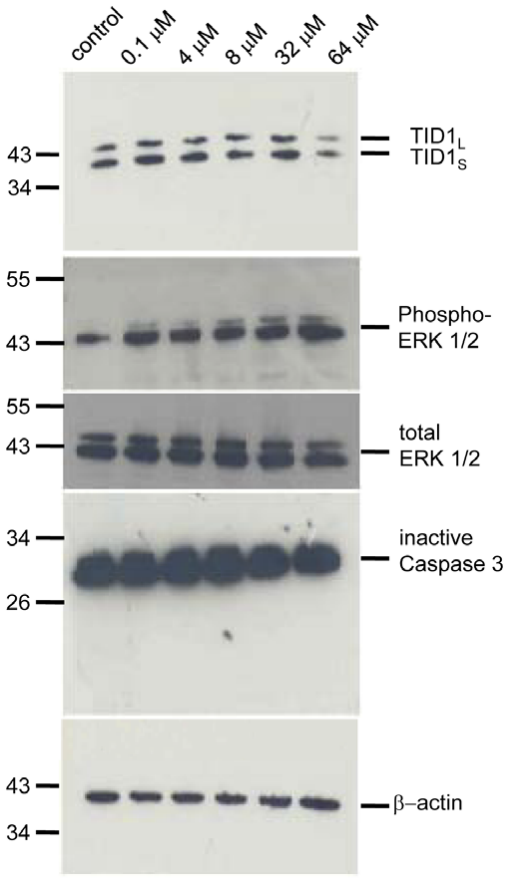
Effect of 6-OHDA treatment for 24 hrs on CAD cells. 40 µg of cell lysate was resolved by SDS-PAGE, transferred to nitrocellulose membrane and stained for TID1, phosphorylated ERK, total ERK, caspase 3 and actin as loading control.

Next we evaluated phosphorylated ERK and total ERK levels in 6-OHDA treated CAD cells (**Figure 9** **;** top panel is from 7A for reference). 6-OHDA-treatment, increased ERK phosphorylation in a dose dependent manner while no change was observed in total ERK expression. This small increase in ERK phosphorylation is not linked to the presence of the 26 kDa TID1 fragment which is not found in toxin-treated neuroblastoma cells. No activation of caspase 3 was detectable following 24 hour 6-OHDA treatment of CAD cells.

In conclusion, members of the J protein family have been proposed to direct client proteins either to maturation or degradation and therefore may be pivotal in controlling the delicate cellular protein folding balance. We speculate that the TID1 quality control system is compromised in PD. Specifically that the 26 kDa TID1 breakdown product plays a role in the pathology of toxin induced Parkinson disease. Widespread presence of the 26 kDa/TID1 entity is documented in the absence of activation of the apoptotic marker caspase 3, corroborating the idea that a specific cellular change outside of the initial site of 6-OHDA application promotes neurotoxicity. Since specific groups of neurons are vulnerable to death in PD, further experimentation is required to address the time frame of cellular TID1 changes in PD, whether cellular TID1 undergoes changes in other experimental models of PD as well as other models of neurodegeneration. The observations reported here are compatible with the notion that strategies to prevent or ameliorate cellular damage in neurodegenerative diseases would ideally target cellular chaperones such as TID1.

## Materials and Methods

### Reagents and Chemicals

6-OHDA and MPP^+^ were from Sigma Aldrich. Anti CSPα polyclonal antibody was prepared as described previously [58]. Anti-Hsp70 mouse monoclonal and anti-Hsp40 rabbit polyclonal were from Assay Design. Anti-Hsc70/Hsp70 mouse monoclonal, anti-β-actin mouse monoclonal and protease inhibitor were from Sigma. 6H4 anti-prion mouse monoclonal was from Prionics, anti-α-synuclein mouse monoclonal was from BD Bioscience, anti-Tid1 mouse monoclonal was from Thermo Scientific Fisher or from Abcam.

### Statement on Animal Welfare

Adult female Long-Evans hooded rats (300–350 g) were raised at the University of Lethbridge vivarium and housed in groups of two in standard polycarbonate shoebox cages. The housing room was maintained on a 12:12 light/dark cycle, with lights on at 7:30 am. The temperature was kept at 22°C and relative humidity at 30%. All procedures were performed according to standards set by the Canadian Council of Animal Care and approved by the University of Lethbridge Animal Welfare Committee (approval ID 1008). A total of 24 rats were used in this study.

#### Nigrostriatal 6-OHDA Lesions

All animals received a unilateral 6-OHDA lesion of the nigrostriatal bundle in the left hemisphere [59]. Thirty minutes prior to surgery, rats received desmethylimipramine (25 mg/kg i.p.; Sigma-Aldrich). The rats were then anesthetized using isoflurane (4% for initiation, 1.5% for maintenance, mixed with oxygen). Neurotoxic lesions of the nigrostriatal bundle were induced by infusing 2 µl of 4 mg/ml (16 mM) 6-OHDA hydrobromide, i.e., in total 30 nmol 6-OHDA, (in 0.02% saline ascorbic acid solution; [12]) at the following coordinates: 4.0 mm posterior to bregma, 1.5 mm lateral to the midline, and 8.5 mm ventral to the skull surface with skull flat between lambda and bregma [60]. Infusion took place over five minutes, with five minutes for diffusion. The skin was then sutured and cleaned, and the animals were allowed to recover on a heating pad before they were returned to their home cage.

### Sensorimotor Behaviour

#### Food Handling Analysis

Abnormalities of posture, limb and mouth movements after 6-OHDA lesion can be observed when rats eat small pieces of uncooked pasta [61], [62]. The motor disability is reflected in the time to eat a piece of pasta. Rats were provided with one 5 cm piece of spaghetti. The time required to eat a piece of spaghetti was recorded, starting with the first contact of the snout with the pasta until the entire piece was consumed and both paws returned to the ground. The following rating scale was used to score the ability to handle and eat the pasta: 0 points were given if the eating time was less than 60 s; 1 point was given if the eating time ranged between 60 and 90 s; 2 points were given if the eating time ranged between 90 s and 120 s; 3 points were given if the eating time exceeded 120 s. This test was repeated three times, separated by a 5 min break each, and averages were calculated.

#### Gait Symmetry Analysis

Gait symmetry and spontaneous turning preference was examined as a predictor of neostriatal dopamine depletion [10], [63]. Following habituation, rats were allowed to explore on an open table top. The ratio of ipsilateral versus contralateral turns was scored using the following rating scale: 0 points if a rat did not show a preference for either side (50/50); 1 point if a rat showed an occasional preference for the ipsilateral-to-lesion side (i.e. 60/40, 70/30); 2 points if a rat showed a frequent preference for the ipsilateral side (i.e. 80/20, 90/10); 3 points if a rat showed absolute preference for ipsilateral side (i.e. 100/0) or the rat was not locomotive and did not turn. The lateral bias of animals was scored for the first ten turns after being placed on the table top. The average was calculated for further analysis.

#### Tactile Stimulation Analysis

Rats with unilateral 6-OHDA lesion show a biased response to tactile stimulation [64]. Following habituation to a tabletop, the rat's vibrissae were stimulated using a Q-tip. The testing side was alternated until 5 trials per side were completed. A score for the affected side was determined according to the following rating scale. 0 points were given if the rat responded to all five contacts; 1 point was given if the rat responded to three or four contacts; 2 points were given if the rat responded to one or two contacts; 3 points were given if the rat does not respond to any contact. The test was repeated twice and average scores were calculated.

#### Statistical Analysis

Statistical analysis was performed using Statview software package 5 for Macintosh (Abacus Concepts Inc.). The results were subject to analysis of variance (ANOVA) for repeated measurements followed by unpaired Student's *t*-tests for comparisons of means and variances between groups. In all statistical analyses, a p-value of less than or equal to 0.05 was considered significant. All data are presented as mean +/− standard error of the mean (SEM).

#### Immunohistochemistry

TID1 was widely detected by immunohistochemistry in frozen PD rat brains. Serial coronal brain sections from the striatum, hippocampus, and substantia nigra were analyzed. Detection of TID1 immuno-reactive cells was enhanced by treating the sections with 50 mM Tris-HCl buffer, pH 9.5, in a microwave oven for 20 min, modified from procedures described elsewhere [65], [66]. Brain sections were incubated overnight in primary antibody mouse monoclonal [RS13] to TID1 (Abcam). Controls were performed omitting the primary antibody. Sections were incubated with a secondary anti-mouse biotinylated antibody (Vector), which was detected using the Elite ABC-peroxidase kit (Vectastain ABC Kit, Vector) with DAB as chromogen (modified from [65], [67]. Sections were examined under light microscopy.

#### Western Blot Analysis

After behavioral tests were completed, animals were deeply anesthetized and decapitated about 27–33 days following the 6-OHDA lesion. The brains were removed, dissected and tissue samples were placed on dry ice. Tissue samples were stored at minus 80°C until further processing. Regions were homogenized in 40 mM Tris (pH 7.4), 150 mM NaCl, 2 mM EDTA, 1 mM EGTA, 0.1% SDS, 1% TritonX100, 0.5 mM PMSF. The total protein concentration was determined using a Bradford reagent (BioRad). 30 µg of homogenate isolated from the indicated brain regions was heated at 95°C for 5 min, resolved by 12% SDS-PAGE, transferred to nitrocellulose and subjected to Western analysis.

#### Cell Culture

CAD mouse neuroblastoma cells [41], [68] were seeded into 6 well plates and grown in DMEM/F12 medium supplemented with 10% fetal bovine serum and 1% Penicillin/streptomycin. *Eahy929* human endothelial cells were seeded into 6 well plates and grown in DMEM medium supplemented with 10% serum, low glucose and hypoxanthine thymidine [69]. Cells were lysed in 40 mM Tris (pH 7.4), 150 mM NaCl, 2 mM EDTA, 1 mM Na_3_VO4, 0.1% SDS, 1% TritonX100, 0.5 mM PMSF and protease inhibitor (Sigma) end-over-end at 4°C for 1 hr. Lysates were centrifuged at 1500xg for 5 min at 4°C and the supernatant was collected. Protein concentration was determined using a Bradford reagent (BioRad). Cell lysate was heated at 95°C for 5 min, resolved by 12% SDS-PAGE, transferred to nitrocellulose and probed with the indicated antibodies.

#### SDS-PAGE and Immunoblotting

Protein samples were separated on 12% SDS-PAGE and transferred from polyacrylamide gels to nitrocellulose (0.45 µm) in 25 mM TrisHCl, 192 mM glycine, 20% methanol and 0.2% SDS for 45 min at 15 V using a Semidry Blotting System (BioRad). Membranes were blocked 4% not-fat dry milk in PBST (PBS, 0.1% (v/v) Tween20) for 30 min at room temperature and probed with primary antibody for 2 hrs at room temperature or overnight at 4°C. The membranes were washed in PBST and incubated with horseradish peroxidase-coupled secondary antibody. The signal was visualized with West Dura Pierce (Pierce Biotechnology, Inc.) using the BioRad Chemi-Doc® System (Biorad) and Hyperfilm (Kodak). The quantification was carried out using QuantityOne (BioRad).
